# Supplementary figures and images for: Construction of ceRNA Networks at Different Stages of Somatic Embryogenesis in Garlic
Source: Int J Mol Sci. 2023 Mar 10;24(6):5311. doi: 10.3390/ijms24065311 (PMC10049443; doi:10.3390/ijms24065311)

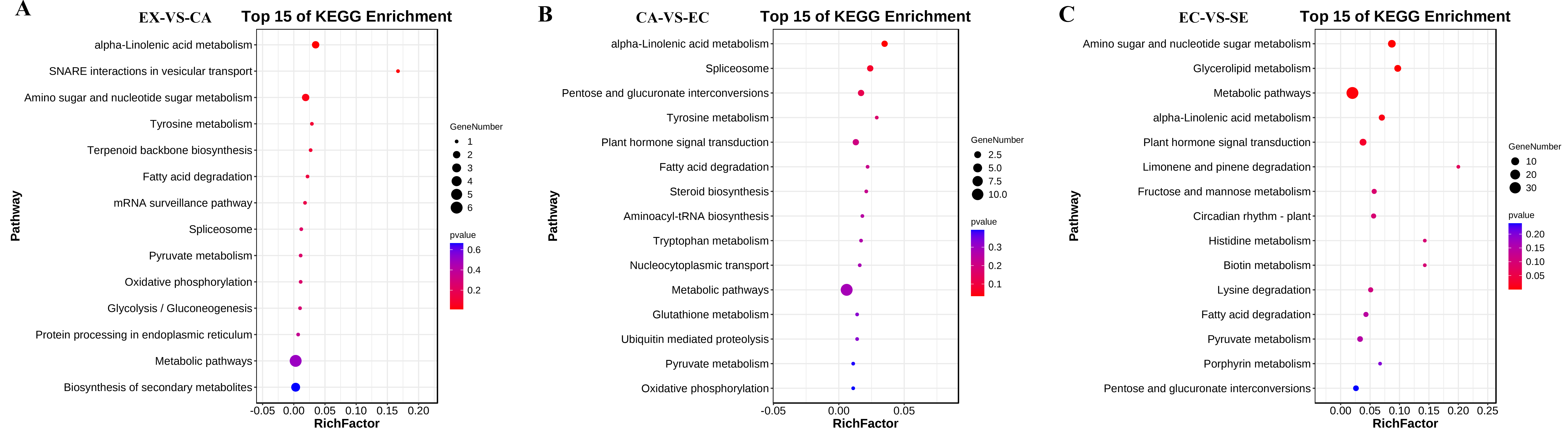

Supplement: Supplementary file 1 [file ijms-24-05311-s001.zip › Figure S3.tif]

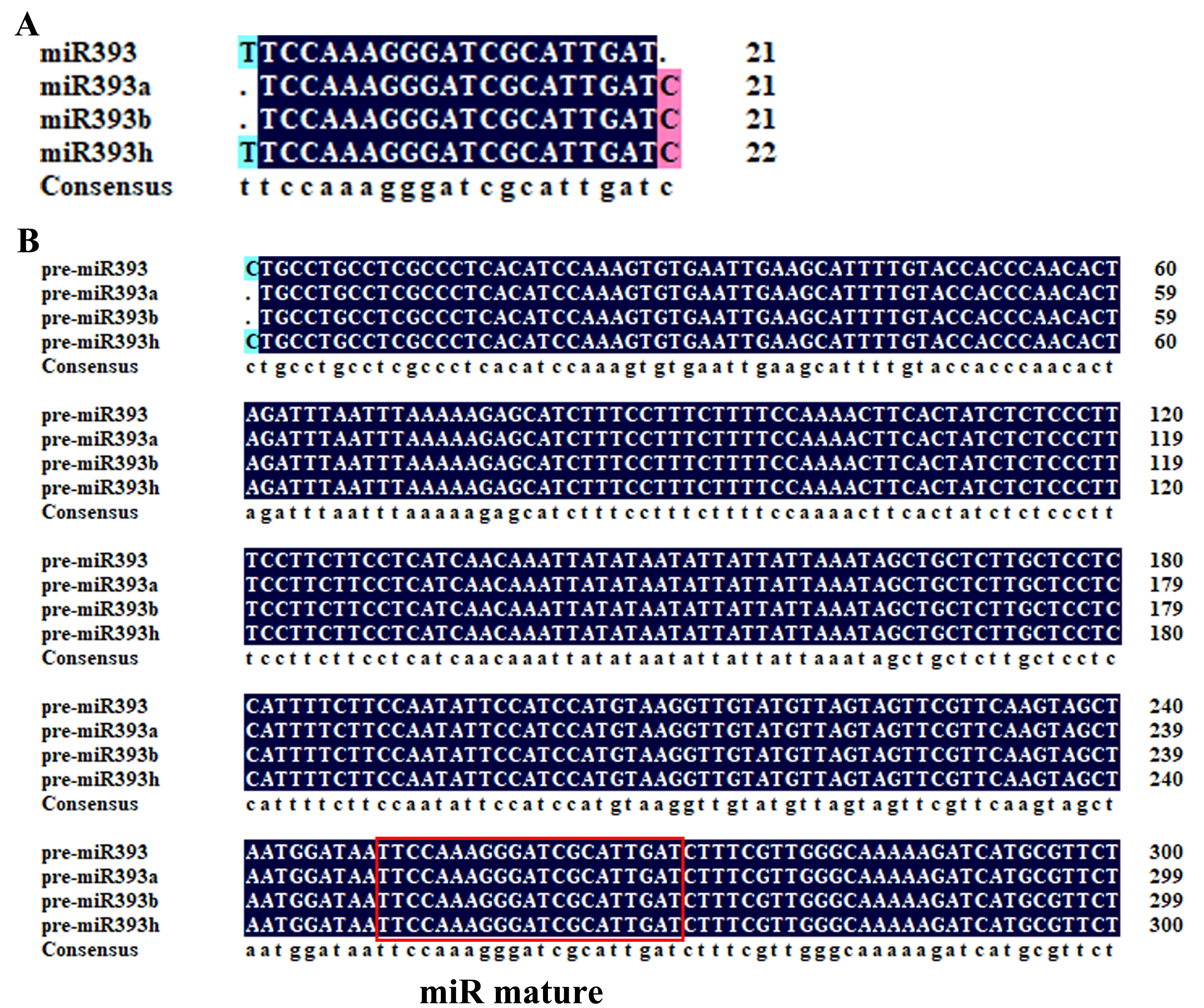

Supplement: Supplementary file 1 [file ijms-24-05311-s001.zip › Figure S5.tif]

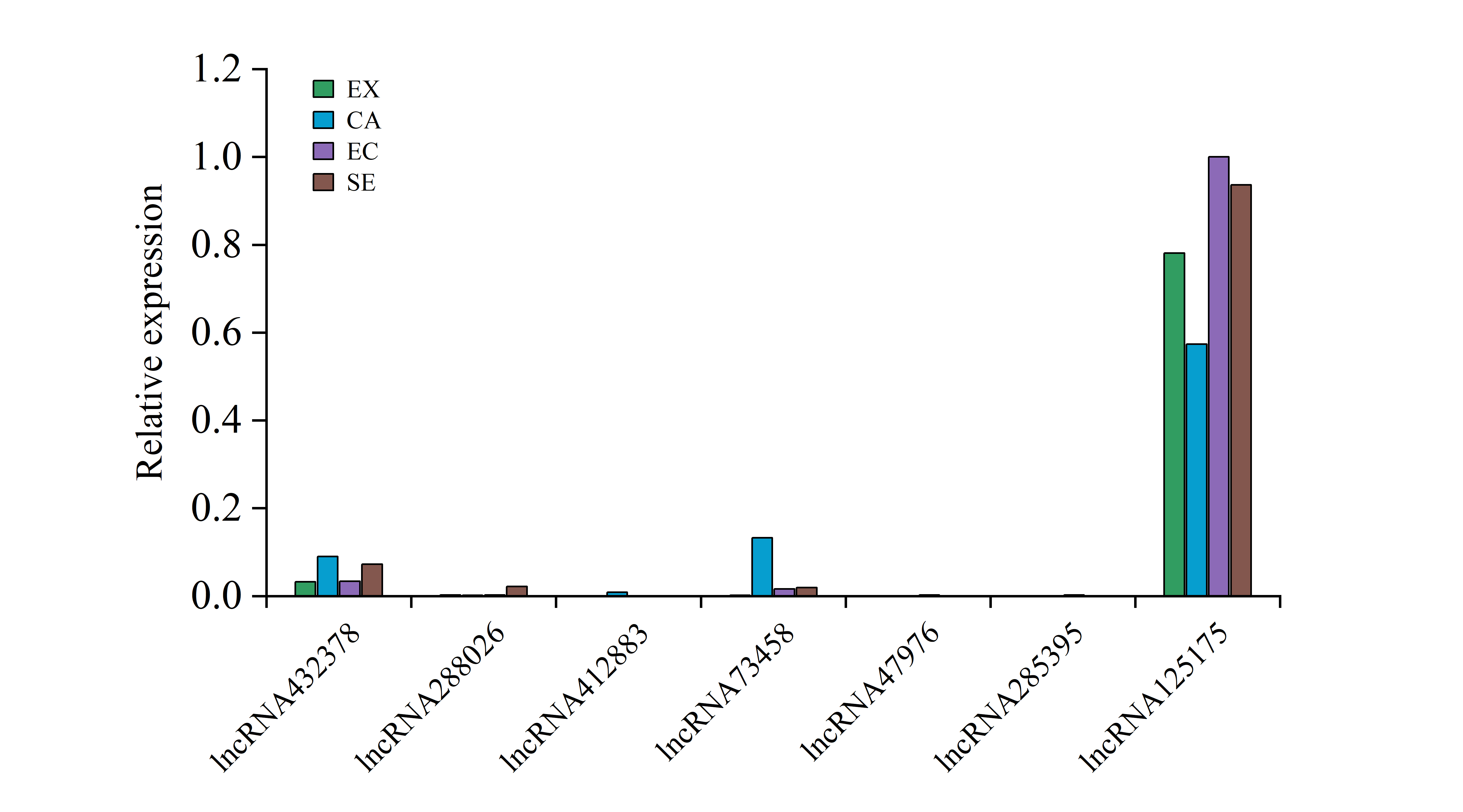

Supplement: Supplementary file 1 [file ijms-24-05311-s001.zip › Figure S6.tif]
